# Parainfluenza Bronchiolitis Mimicking Recurrent Lobar Pneumonia

**DOI:** 10.7759/cureus.26818

**Published:** 2022-07-13

**Authors:** Keshav Bhattar, Trupti Pandit, Ramesh Pandit

**Affiliations:** 1 Internal Medicine, Dr SN Medical College, Jodhpur, IND; 2 Hospital Medicine/Pediatrics, Inspira Medical Center, Vineland, USA; 3 Medicine, Independent Researcher, Philadelphia, USA; 4 Hospital Medicine, University of Pennsylvania/Chester County Hospital, Philadelphia, USA

**Keywords:** hpiv, length of stay, recurrent hospitalization, geriatric, mucus plug impaction, lobar pneumonia, recurrent pneumonia, bronchiolitis, parainfluenza

## Abstract

Human parainfluenza viruses (HPIVs) are the second most common cause of hospitalization in children, causing upper respiratory tract illness (URTI) and lower respiratory tract illness (LRTIs) in infants and young children. Common presentations include common cold, laryngotracheobronchitis (croup), bronchitis, and pneumonia. In immunocompetent adults, their effect is usually limited to mild upper respiratory tract illness with spontaneous recovery. However, elderly and immunocompromised adults are at risk for severe infection manifesting as epiglottitis, bronchiolitis, pneumonia, and on rare occasions, acute respiratory distress syndrome (ARDS). We describe a case of a 73-year-old female who developed recurrent respiratory distress and acute hypoxemic respiratory failure and was treated for bacterial pneumonia but was eventually diagnosed with severe parainfluenza bronchitis, causing mucus plug obstruction and lobar lung collapse.

## Introduction

Parainfluenza viruses (PIVs) are single-stranded, positive-sense, linear ribonucleic acid (RNA) viruses belonging to the family Paramyxoviridae. Human PIVs (HPIVs) are classified into five subtypes: HPIV-1, HPIV-2, HPIV-3, HPIV-4a, and HPIV-4b. HPIV-1 and HPIV-2 are associated with croup. HPIV-4 causes less severe disease. HPIV-3 is the most virulent of the group, causing significant morbidity and mortality. HPIV infection mortality is low in developed countries, except in young infants, elderly and immunocompromised patients. In developing countries, pre-school children are at increased risk, with super-added bacterial infections contributing notably [1].

Presentation typically includes a history of coryza, low-grade fever, barking cough, pharyngeal erythema, and inspiratory stridor. The subglottic edema frequently presents in children as partial upper airway obstruction and subsequent stridor, characteristic of croup. Subglottic swelling and narrowing from the involvement of the larynx and trachea may be visualized as the classic ‘steeple sign’ on an anteroposterior neck x-ray. In adults, they can cause community-acquired respiratory tract infections of varying severity. There is an increased risk of reinfection in elderly and immunocompromised patients. It is frequently implicated in acute exacerbations of asthma and chronic obstructive pulmonary disease [2]. People living in long-term nursing facilities are at increased risk. Systemic flu-like symptoms are more common in adults. Radiographic findings commonly include unilateral or bilateral infiltrates, with more than half of the cases demonstrating concurrent bacterial infection [1].

## Case presentation

A 73-year-old female presented to the emergency department with a four-day history of cough, shortness of breath, and fever (T-max of 102). Cough was associated with non-malodorous thick yellow secretions and posterior oropharyngeal erythema (concerning for viral process). She also complained of throat swelling and difficulty swallowing. Dyspnea was associated with orthopnea and hypoxemia (pulse oximeter at home gave a reading of 88% on room air). She did not report any chest pain, tightness, night sweats, or unintended weight loss.

She has a past medical history of chronic kidney disease (stage IIIA), partial left nephrectomy one year ago for angiomyolipoma (likely papillary renal cell carcinoma per path report), bilateral renal cysts, and post-traumatic stress disorder, chronic constipation, and Takotsubo cardiomyopathy.

On admission, the complete blood count (CBC) was within normal. Brain natriuretic peptide (BNP) levels were within the normal range. Chest x-ray revealed subtle opacity at the right lung base, and Parainfluenza serology was positive. She was started on intravenous azithromycin and ceftriaxone for presumed secondary bacterial pneumonia. Inhaled fluticasone and ipratropium-albuterol were also started to help her respiratory distress.

She required 3-4 liters per minute of oxygen (O2) supplementation throughout her four days of hospitalization and attempts to wean her off were unsuccessful. Hence, she was discharged with home O2 at 2 liters per minute, fluticasone, and ipratropium-albuterol for home therapy, with oral amoxicillin-clavulanate to complete five days of the antibiotic course. 

Hospitalization 2

After 10 days, she presented again with dyspnea, increasing cough, and worsening intermittent hypoxia. The characteristics of the dyspnea were the same as her last admission, with the notable absence of fever. Testing for infectious etiology was negative except for the persistence of Parainfluenza type 3 (both in serum and bronchoalveolar lavage). She was started on intravenous cefepime for concern of persistent bacterial pneumonia.

**Figure 1:**
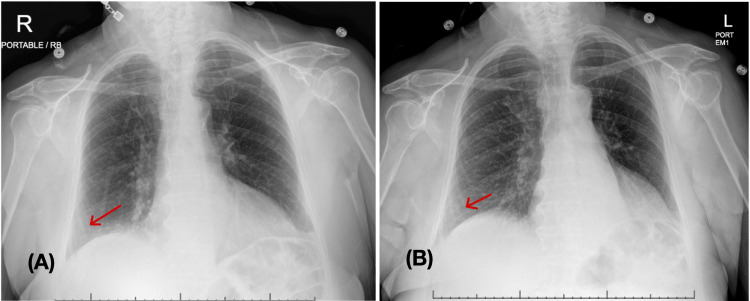
Chest x-ray anterior and posterior (AP) view (A) Left: Prior admission 10 days ago - subtle opacity at the right lung base; (B) Right: latest admission - right basilar opacities have slightly improved, no other significant interval change

**Figure 2:**
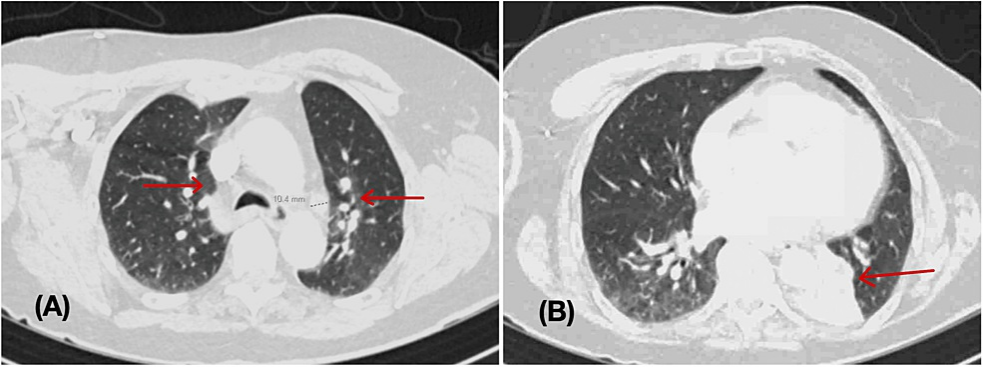
CT pulmonary angiogram (A) 2 x 2 cm left hilar nodular soft tissue lesion, calcified hilar lymph nodes with new lymphadenopathy (right hilar, paratracheal), and bilateral bronchial wall thickening.  Multiple scattered nodules as elongated/spiculated 1.1 cm at the left lower lobe (Arrows). No evidence of pulmonary embolism. (B) - cardiomegaly with mild pericardial effusion, bibasilar reticulonodular opacities. Filling defects in the adjacent left mainstem bronchus with complete impaction of medial left lower bronchi and associated lung collapse (arrow).

**Figure 3:**
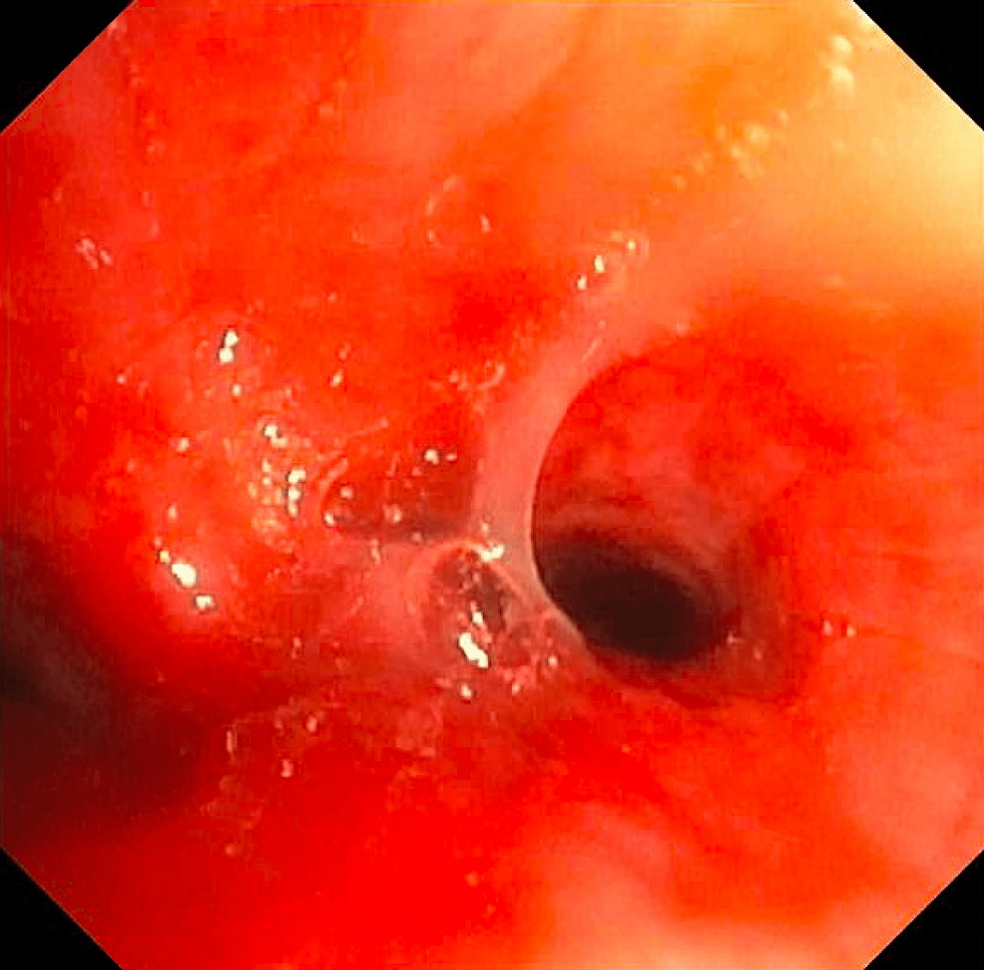
Bronchoscopy The right and left bronchial trees with thick inspissated secretions; white in color, and severe mucosal inflammation with easy bruising with suctioning and scope trauma. Upon suctioning of secretions, no endobronchial lesions were seen.

Chest x-ray showed no significant change from the previous imaging (Figure 1). D-dimer was elevated at 1.03 ug/mL (reference range <0.49 ug/mL). Hence, a computed tomography (CT) angiogram of the chest was ordered, which showed no definite evidence for pulmonary embolism but revealed a collapsed left lower lobe of the lung with associated obstructing hilar lesion, lymphadenopathy, and new lower predominant reticular nodular opacities (Figure 2). The differential diagnosis at this point included metastatic disease (possibly from a recurrence of her renal carcinoma) or an infectious/inflammatory etiology. She underwent bronchoscopy, which revealed thick, clear, inspissated secretions with severe mucosal inflammation as the cause of left lower lung lobe atelectasis (Figure 3). She was started on high-dose inhaled corticosteroids (fluticasone 220 mcg/puff; four puffs TID with spacer) for severe coughing and bronchitis. 

She continued to have orthopnea with the development of trace bilateral pedal edema. Her BNP level was elevated at 184 pg/mL. Because of her history of Takotsubo cardiomyopathy, a cardiac echocardiogram was ordered, which showed a left ventricular ejection fraction (LVEF) of 50-55% with inferior, inferoseptal, and inferolateral wall hypokinesis. Prior imaging was not available for comparison. Meanwhile, she had some symptomatic relief for congestion and cough with guaifenesin-codeine and acetylcysteine. Her dry coughing spells persisted (likely due to post-viral bronchitis). Oral prednisone 20mg/day was added to his inhaled fluticasone regimen. Chest physiotherapy and incentive spirometry were started. However, the cough was still present. She desaturated to 90% on room air. She continued to have difficulty sleeping because of the inspissated mucus plugs and reported substantial relief with chest physiotherapy and mucolytic medications.

On day 14 of hospitalization, she was discharged on oxygen (with a home oxygen concentrator) for ambulation based on an updated home oxygen assessment (oxygen saturation [SpO2] of 92% at rest in room air, 84% on exertion in room air, and 93% on exertion with 2 liters per minute) with advice to follow up with her pulmonologist in two weeks. A vibratory vest was also advised to help with the persisting mucus plugs. Prednisone was continued at 20mg daily for a total of 10 days (Table 1).

**Table 1:** Lab test results RNA - Ribonucleic acid, NAAT - Nucleic Acid Amplification Test, RT-PCR - Reverse transcription Polymerase Chain Reaction

TEST	First Hospitalization	Second Hospitalization (ten days later)
Blood culture and sensitivity (FINAL)	No growth	No growth
Urinalysis	Within normal limits	Within normal limits
COVID 19 Accula RNA NAAT	Not detected	Not detected
INFLUENZA A, RNA RT-PCR	Not detected	Not detected
INFLUENZA B, RNA RT-PCR	Not detected	Not detected
Legionella antigen, Urine	Not detected	Not detected
S. pneumoniae Antigens, Urine	Not detected	Not detected
Mycology Direct Exam and Culture	No yeast or fungi were seen. No growth in culture.	No yeast or fungi were seen. No growth in culture.
Serum respiratory pathogen panel (RT-PCR)	Parainfluenza type 3	Parainfluenza type 3

## Discussion

Bronchiolitis is an injury to the bronchioles (smaller airways of 2 mm or less). Primary bronchiolitis can be classified as acute bronchiolitis (caused by viral infections, mainly seen in infants and children), respiratory bronchiolitis (most common form in adults and is usually related to cigarette smoking), constrictive (obliterative) bronchiolitis (environmental/occupational inhalation exposures, transplant recipients (bronchiolitis obliterans syndrome), diffuse idiopathic pulmonary neuroendocrine cell hyperplasia (DIPNECH)), diffuse aspiration bronchiolitis, diffuse panbronchiolitis, follicular bronchiolitis, or mineral dust airway disease. Bronchiolitis is also associated with interstitial lung diseases and disorders of large airways like asthma, chronic bronchitis, or bronchiectasis [3]. Bronchiolitis obliterans organizing pneumonia (BOOP), which is now called “organizing pneumonia” (OP), manifests as parenchymal lung disease rather than strictly bronchiolar disease.

Pathogenesis of bronchiolitis due to HPIV is similar to a respiratory syncytial virus (RSV), where lower respiratory tract infection predisposes to episodic bronchoconstriction and hypoxemia. On chest CT scans, viral bronchiolitis manifests as centrilobular nodules and multifocal “tree-in-bud” opacities rather than bronchial wall thickening (bronchitis) or consolidative/ground-glass opacities (pneumonia) [4]. It is also common to see excessive mucus production in bronchiolitis, secondary to goblet cell hyperplasia, which is triggered by the inflammatory response. However, atelectasis resulting from mucus build-up from an inflamed lower respiratory tract is very uncommon. RSV has been shown to increase airway mucus secretion by down-regulation of miR-34b/c-5p expression in airway epithelial cells (miRNA, a negative regulator of gene expression) [5]. HPIV on the other hand has not been studied rigorously in this regard.

HPIV transmission occurs via large aerosol droplets or direct inoculation through contaminated fomites. Our patient also had a strong history of contact with possibly contagious people. Peak inflammatory response in the airways occurs seven to 10 days after initial exposure, manifesting as airway inflammation, necrosis and sloughing of the respiratory epithelium, edema, increased mucus production, and interstitial infiltration of the lung. Along with anterior posterior (AP) neck and chest x-rays, lateral neck x-rays are also helpful to assess signs of foreign body aspiration, retropharyngeal abscess, or epiglottitis (which may be visualized as the classic ‘thumb sign’ due to enlargement of the epiglottis and ballooning of the hypopharynx). Complications include acute respiratory distress syndrome (ARDS), fatal pneumonia, and rare complications, including Guillain-Barré syndrome [6] and meningitis [7]. Post hematopoietic stem cell transplant (HSCT) HPIV infection frequently manifests as serious lower respiratory tract disease in adults and children who undergo bone marrow transplants (BMT).

It has been suggested that viral detection in the lungs of infected patients is associated with worse outcomes when compared with virion detection in the upper respiratory tract. This can be used as a predictor of poor outcomes [8]. Our patient also had HPIV detected in her bronchoalveolar lavage sample, which may have contributed to her prolonged hypoxemia.

Immunity after primary HPIV infection usually elicits a decent antibody response. However, reinfection can occur multiple times throughout life, even in the presence of neutralizing antibodies [9]. Most of these reinfections are limited to the upper respiratory tract. Nevertheless, severe lower respiratory tract involvement is frequently seen in immunocompromised states (most notably bone marrow transplant and primary immunodeficiencies). Our patient had chronic kidney disease (stage III-A), which diminishes innate and adaptive immune responses [10]. In a British retrospective cohort study of 191,709 patients, McDonald et al. reported that compared with patients with estimated glomerular filtration rate (eGFR) ≥ 60 mL/min/1.73 m2, those with eGFR < 15 or 15 to 29 had adjusted incidence rate ratios of 3.04 and 1.73 respectively for pneumonia. History of proteinuria was reported to be an independent marker of increased infection risk for lower respiratory tract illness (LRTI), pneumonia, and sepsis [11]. However, our patient’s GFR ranged from 57-75mL/min/1.73 m2, and her urinalysis showed no signs of proteinuria during her hospitalizations.

Steroids and cool oxygen mist are the most commonly used therapies for symptomatic management. A single dose of oral dexamethasone in the office or emergency room provides relief by reducing edema. Intramuscular dexamethasone or budesonide may be used in patients who are vomiting or in severe respiratory distress. For patients requiring escalation of therapy, administering racemic epinephrine with a nebulizer has been beneficial. Endotracheal intubation followed by a tracheotomy may be indicated if there is severe airway obstruction due to nasopharyngeal swelling. Ribavirin is a broad-spectrum antiviral effective against HPIV-3 infection [12]. However, most of the data regarding the role of ribavirin come from case reports or case series of immunocompromised patients (primary immunodeficiencies, HSCT, and solid-organ transplant recipients). The effectiveness of ribavirin has not been consistently reproduced even in this population subgroup. Nichols et al. reported that aerosolized ribavirin with or without intravenous immunoglobulin (IVIG) did not alter mortality or decrease the duration of HPIV viral shedding in recipients of HSCT [13]. Hence, its efficacy in adult immunocompetent patients has not been studied robustly. For such patients, IVIG [14] and a novel sialidase fusion protein inhibitor, DAS181 [15], have also been used, but further study is needed. While severe RSV bronchiolitis has been successfully treated with RSV-IVIG and palivizumab (humanized murine monoclonal IgG1 antibody against the RSV F-glycoprotein, given as a monthly intramuscular injection during the RSV season), HPIV infections have not yet been treated by targeted therapies against its virion.

Currently, there are no licensed vaccines for the prevention of infections by HPIVs though the research is ongoing. Most efforts are currently being made to develop a vaccine against HPIV-3. However, the cross-protection offered by an HPIV vaccine of a particular serotype is minimal or short-lived, making multiple or multivalent vaccines necessary. As RSV and HPIV affect the same age groups, recombinant vaccines that express both RSV and HPIV proteins are being developed [9]. Recent studies in the early phase trials have shown the development of neutralization titers against HPIV with mRNA-based vaccines, with the possibility of annual vaccination requirements [16].

## Conclusions

HPIV is a common cause of both upper and lower respiratory tract infections in children and the immunocompromised. However, in immunocompetent adults, the disease is usually limited to mild-to-moderate upper respiratory tract infections. Bronchitis and bronchiolitis caused by HPIV infection can cause excessive mucus production, which may progress to lobar or lobular atelectasis, resulting in recurrent acute hypoxemic episodes and nocturnal dyspnea and orthopnea. This presentation may be incorrectly diagnosed as lobar pneumonia, especially if there is an accompanying fever. Bronchoscopy is essential to rule out more sinister pathologies that may cause segmental lung collapse. Steroids are usually effective and are often given more importance than adequate mucolytic therapy. Chest physiotherapy and mucolytics play a crucial role in management. Given the changing disease burden among viral respiratory infections, developing vaccines against HPIV must be considered aggressively for prevention, with mRNA-based vaccines showing promise.
